# Sensory Profile of Chihuahua Cheese Manufactured from Raw Milk

**DOI:** 10.1155/2018/8494105

**Published:** 2018-12-23

**Authors:** Sarai Villalobos-Chaparro, Erika Salas-Muñóz, Néstor Gutiérrez-Méndez, Guadalupe Virginia Nevárez-Moorillón

**Affiliations:** Facultad de Ciencias Químicas, Universidad Autónoma de Chihuahua, Chihuahua, 31125, Mexico

## Abstract

Chihuahua cheese is a local artisanal cheese traditionally produced from raw milk. When this cheese is produced with pasteurized milk, cheesemakers complain that there are differences in taste and aroma as compared with traditional manufacturing. This work aimed to obtain a descriptive sensory analysis of Chihuahua cheese manufactured with raw milk under traditional conditions. Samples were collected in five cheese dairies at two different seasons (summer and autumn), and a Quantitative Descriptive Sensorial Analysis was done by a panel of trained judges. For aroma descriptors, cooked descriptor showed differences between dairies, and whey was different among dairies and sampling seasons (P<0.01); diacetyl, fruity (P<0.01), as well as free fatty acids, nutty and sulphur (P<0.05) descriptors varied between seasons. For flavour descriptors, bitter perception was different between dairies and seasons (P<0.01). Salty and creamy cheese was also different among dairies (P<0.01). A Principal Component Analysis for differences among dairies and sampling season demonstrated that the first three components accounted for 90% of the variance; variables were more affected by the sampling seasons than by the geographical location or if the dairy was operated by Mennonites. Chihuahua cheese sensorial profile can be described as a semi-matured cheese with a bitter flavour, slightly salted, and with a cream flavour, with aroma notes associated with whey and sour milk. Principal Component Analysis demonstrated season influence on flavour and aroma characteristics.

## 1. Introduction

Cheese has been produced for centuries, using the milk of many domestic animals, including cow, sheep, and buffalo. There are many types of cheese, all of them have been initially manufactured within a particular human community where a specific cheesemaking process was developed. Traditional cheese products are characterized by diversity in their manufacturing processes, and when raw milk is used, a rich and diverse microbiota is associated with its production. Traditional cheeses need to be studied considering the geographic location, history, and other social variables of the human communities where they are produced. Also, climate, milk characteristics, microbiota associated with cheese factories, and other environmental conditions contribute to specific and particular flavour and aroma of each product [1, 2]. In order to protect and preserve the production of traditional food products, certifications such as Protected Designation of Origin (PDO) and Protected Geographical Indication (PGI) have been awarded to food products that demonstrate their differences and particular social and cultural conditions of production. In Mexico, many traditional types of cheese have been elaborated for centuries with raw milk, but foodborne outbreaks associated with dairy products have led to mandatory use of pasteurized milk in the production of most cheese types, except some PDO aged cheeses (minimum six months of ripening period) [3].

Chihuahua cheese manufactured with unpasteurized milk has been produced for almost a century in the northern state of Chihuahua, Mexico, and is the result of the interaction between the Mennonite community and local farmers [4]. Chihuahua cheese manufacturing is similar to Cheddar cheese, but it is usually consumed without maturation; it is then considered as a young semi-hard cheese elaborated with cow milk [5]. It is usually consumed within weeks of manufacturing and is usually not accepted by local consumers after a few months of maturation. Nowadays, different products are identified as Chihuahua cheese, including traditional cheese manufactured by the Mennonite community using either pasteurized or unpasteurized milk; traditional cheese manufactured by non-Mennonite farmers, as well as industrialized cheese, was elaborated outside Chihuahua state or even outside Mexico [6]. Chihuahua cheese manufacturing process has been reported previously, including time and temperature conditions throughout processing. A considerable variation in manufacturing conditions has been observed, due to nonstandardization of the process [7].

There have been few efforts to characterize and define the particular flavour and aroma characteristics of Chihuahua cheese, including cheese produced in Chihuahua state [5, 8, 9] as well as Chihuahua-type cheese [10]. Almanza-Rubio et al. [6] demonstrated in a study with consumers from the state of Chihuahua that there is a preference to consume cheese elaborated in Chihuahua State as compared with Chihuahua-type cheese elaborated elsewhere.

The traditional manufacturing process used raw milk as starting material, but because of government regulations [11], pasteurization has been included in the process, although cheesemakers claim that there are differences on flavour and aroma after milk pasteurization. In order to propose the use of pasteurised milk for the artisanal manufacturing of Chihuahua cheese, State government has an intensive training program in good manufacturing practices, as well as support to improve infrastructure in small and medium-size cheesemaking facilities. Therefore, it is essential to describe the sensorial profile of Chihuahua cheese manufactured with raw milk, to serve as a reference for cheese produced with pasteurised milk and with the addition of starter cultures.

Therefore, this work aimed at providing information for the development of a sensorial profile of Chihuahua cheese manufactured with raw milk, as well as to describe the effect of season and area of food production.

## 2. Materials and Methods

### 2.1. Chihuahua Cheese Samples

In order to obtain a complete description of the sensorial characteristics of Chihuahua cheese elaborated by Mennonites and non-Mennonites farmers, five dairies located in the state of Chihuahua, Mexico, were selected for this study. Two locations were owned and operated by Mennonites (cheese dairies B and C) and three by non-Mennonites (A, D, and E). Dairies elaborated artisanal Chihuahua cheese using raw milk and without the addition of a starter culture; samples were taken during summer and autumn 2013, according to the procedure described in Sánchez-Gamboa et al. [7]. For each season, one block of cheese (1-2 kg) was obtained from the dairies and transported to the laboratory within 24 h of manufacture. In order to complete the initial maturation process used by cheesemakers, cheese blocks were stored at 18°C for three days. Cheese portions (250 g) were vacuum-packed using a commercial system, including their trademarked vacuum bags (Food Saver V2830, Sunbeam Products, Oklahoma City, USA) and stored at 6.5°C in a container under dark controlled conditions for three months before sensory analysis.

### 2.2. Panel Selection and Quantitative Descriptive Analysis of Chihuahua Cheese

After an initial selection from 30 university members [12], eight were included in the panel. Panel members (2 male, 6 female, ages 24-32) were further trained (minimum 75 h) using the SpectrumTM 15-point intensity scale [12] for cheese flavour and aroma, based on descriptors suggested by Drake et al. [13, 14] for Cheddar cheese. During training, panelists have presented references, as indicated in Table 1 as well as Chihuahua cheese samples for the identification of descriptive flavour and aroma terms. Descriptors related to aroma terms were evaluated by nasal perception.

Cheese samples were cut into 2 cm by side, tempered for 30 minutes to 20 (±2) °C before evaluation, and presented in plastic cups with a randomly assigned code. For training purposes, panelists received information on Chihuahua cheese manufacturing, and the team evaluated and discussed their evaluation of cheese samples used for training, in order to minimize variability. Sensory evaluations were done in a sensory lab, with environmentally controlled booths and white lights. Sensory evaluations were carried out when cheese samples were stored for three months; therefore, evaluations of summer and autumn samples were done at different times. In each session, panel members evaluated three cheese samples; salt crackers and coffee beans were provided for the panelists to eliminate residual flavour and odour between each sample analysed. Data were collected on a paper ballot, with a nine-point scale.

### 2.3. Statistical Analysis

Data collected were subjected to analysis of variance (Block Full Factorial ANOVA design) for each sensorial parameter, using production area and sampling season as independent variables, and panelists were considered as blocks for a Complete Block Design. Tukey means analysis was used to differentiate groups, using a 5% significance level. Mean values obtained for each sensorial parameter were used for Quantitative Descriptive Analysis to generate radar charts for each dairy. A Principal Component Analysis (PCA) and a K-means clustering were also carried out to evaluate if there was a relationship between individual cheese samples and dairy or sampling season. In the graphic presented, samples are observed as dots [15]. Statistical analysis was done using the statistical software Minitab 17 [16].

## 3. Results

The descriptive analysis of Chihuahua cheese was based on descriptors used by Drake et al. [13] (Table 1) that were used to train a sensorial panel composed of eight members. Mean score values for each descriptor are summarized in Table 2; statistical analysis was done to determine differences between dairies, sampling period, and the interaction of those two variables; the table also includes a summary of the results of analysis of variance for each descriptor.

Based on the statistical analysis (Table 2), the aroma descriptor “cooked” showed difference between dairies (P<0.01) being dairy B the one with the highest mean score (2.9). The aroma descriptor “whey” showed a highly significant difference for both, the dairy where the cheese was produced and the sampling season (P<0.01). Cheese sampled during summer had the highest value for this parameter (3.7 versus 2.9 for autumn), and dairy A obtained the highest value (4.2). The diacetyl aroma perceived by the sensory panel was different between seasons (P<0.05), but not among dairies; the same pattern was observed in the fruity (P<0.01), free fatty acids (P<0.05), nutty (P<0.05), and sulphur (P<0.05) descriptors of aroma.

For the flavour descriptors, bitter perception was statistically different between dairies ((P<0.01) (A, B > C > D, E) as well as between seasons (P<0.01) (Summer 3.8, Autumn 2.7). Regarding the perception of salty in cheese, differences were observed between dairies ((P<0.01). The creamy descriptor, which can be related to the highest concentration of milk fat in this dairy product, was perceived different among the dairies analysed (P<0.01) (C > B, D > A > E) but not among sampling seasons. No differences were presented for the sweet and umami descriptors among dairies or sampling season.

The descriptors that presented a significant interaction between dairy and sampling season are indicated with asterisks in Table 2. Cheese from dairy A sampled during summer had the highest scores for whey, diacetyl, free fatty acids, bitter, and salty terms. In general, the highest scores were presented for Chihuahua cheese manufactured during summer. The terms with the highest scores were bitter, creamy, and whey.

**Figure 1:**
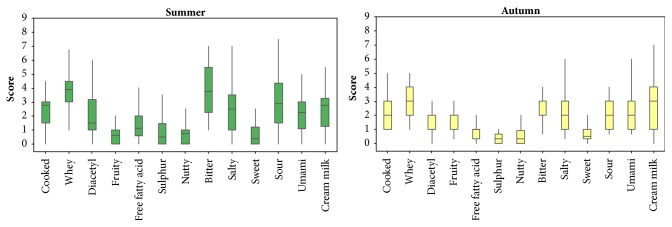
Boxplot of Quantitative Descriptive Analysis of Chihuahua cheese manufactured with raw milk and sampled during summer and autumn. Median, 1^st^ and 3^rd^ quartile, minimum, and maximum scores are presented for each descriptor.

The effect of season on the flavour and aroma of Chihuahua cheese can be better observed in Figure 1, where the median, quartiles, and minimum and maximum score values for each descriptor are incorporated in a boxplot graphic, separated by season. Although in both seasons the descriptors with the highest scores are the same, score values for the cheese manufactured during autumn have lower values than those manufactured in summer, except for the fruity descriptor.

**Figure 2:**
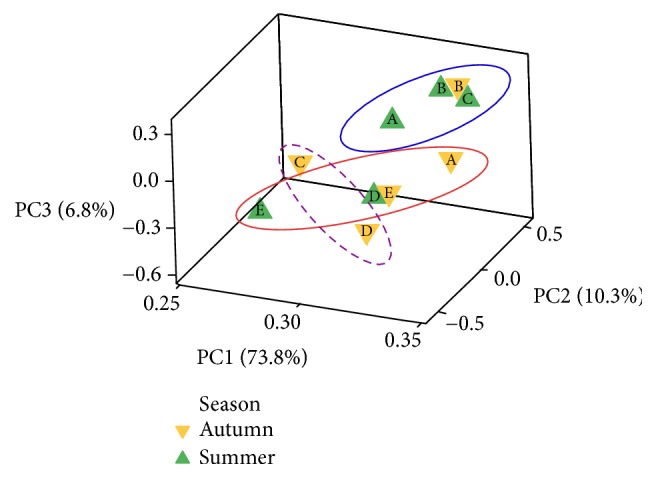
Principal Component Analysis for dairies and sampling period of Chihuahua cheese for sensory analysis. Scores of each sample for the three first Principal Component Analyses (PCA) are shown.

In order to relate all descriptors used in the sensorial analysis of Chihuahua cheese with seasonality and dairy analysed, a PCA was done using dairies and seasons as independent variables. The first three components explained 90.9% of data variation; PC1 accounted for 73.8% of the variation, while PC2 explained 10.3% and PC3 6.8% of variance.  Figure 2 shows the position of each of the dairies in each season related to the three first components of the analysis. PC1 has an almost homogeneous positive load in all cheese dairies and sampling seasons (coefficient values 0.25-0.34), while PC2 has a positive correlation with cheese manufactured in dairy C (non-Mennonite owner) during autumn (0.539), and a negative correlation with cheese from dairy E, also from non-Mennonite owner produced in summer (-0.562). PC3 coefficients were positive for Mennonite owned dairies and were negative correlated to cheese produced in autumn from dairies C and D (-0.50 and -0.60, respectively).

K-means analysis identified three groups that are more related to the season of sampling than to dairy or even geographical location of each farm. One of the groups included dairies A and E sampled during autumn, as well as summer dairies D and E. Another group included cheese samples from dairy B autumn, and dairies A, B, and C summer. The third group identified included autumn samples from dairies C and D.

## 4. Discussion

### 4.1. Aroma and Flavour Descriptors for Chihuahua Cheese

In order to generate a sensorial profile of Chihuahua cheese manufactured with raw milk, the aroma and flavour descriptors proposed by Drake et al. [13] for Cheddar cheese were used in this study. The term creamy was also added, based on previous analysis (data not published).

Aroma descriptors included the terms cooked, whey, diacetyl, fruity, free fatty acid, sulphur, and nutty. The last two terms obtained low scores by the sensory panel; therefore, they are not related to specific aroma notes in Chihuahua cheese. “Sulphur” aroma has been associated with Cheddar cheese [13], related to the release of sulphur compounds from amino acid breakdown, and is commonly found in riped cheeses [17]. The term “nutty” is given by volatile compounds also derived from amino acid metabolism that have been reported in Emmental or Gruyere cheese [18]. Such cheese types are also consumed after long ripening.

The aroma terms of “diacetyl”, “free fatty acids”, and “fruity” are related to the metabolism of fatty acids, either by auto-oxidation to give the aroma related to butter, the release by lipolysis of volatile fatty acids such as butanoic, hexanoic, or octanoic acid, or fruity notes derived from esterification reactions of alcohol and fatty acids [19]. The notes related to fruits have been associated to Italian cheese, where they balance the presence of free fatty acids [20] and have also been associated with Cheddar, as well as with the Italian cheeses, Grana Padano, and Ragusano [21, 22]. These three terms were identified as different between sampling seasons, but not between dairies, which can be related to the difference in milk composition due to variation in the composition of the cow's milk through the year.

The term “cooked” is related to the presence of compounds derived from the Maillard reaction and is expected to be higher in cheese manufactured with pasteurized milk; although all cheese samples studied in this work were elaborated with raw milk, differences among dairies can be related to physicochemical characteristics of the milk used or to differences in processing conditions [7]. The term “whey” relates to the smell of fresh whey developed during cheese production that contains sugar, proteins, and fatty acids not retained in the curd [23]. Mean scores for this descriptor had a high mean score in the samples analysed; therefore, this descriptor can be associated with Chihuahua cheese. The term was also different among dairies and seasons, so is one of the most important aroma descriptors related to the differences in Chihuahua cheese production. The differences can be related to the milk composition and the manufacturing processes.

Flavour descriptors evaluated included the five basic tastes and creamy. The term “bitter” can have different sources related to their perception including ripening time, microorganisms present and their metabolism, or milk composition. This term had a high mean score in Chihuahua cheese, as well as differences among dairies and seasons. Chihuahua cheese was also identified as “salty” by the sensory panel; differences among dairies can be related to differences in traditional manufacturing of Chihuahua cheese [7]. “Cream-like” flavour is related to fresh cheese types rather than to ripped ones. Chihuahua cheese has a high score for creamy that is influenced by the dairy and by the season of manufacturing.

“Sweet” and “umami” descriptors did not show statistical differences among dairies or between sampling seasons; “sour” descriptor was different among seasons (Table 2), and Chihuahua cheese had relatively high mean scores for sour and umami terms. The umami descriptor is related to glutamate presence, and the concentration of this aminoacid increases during ripening. Aged Cheddar cheese has been reported to increase tenfold the concentration of glutamate after eight months of ripening [24].

### 4.2. Quantitative Descriptive Analysis (QDA)

Differences for the descriptors between seasons can be observed in Figure 1, which summarizes the QDA analysis of Chihuahua cheese included in this study. Chihuahua cheese can be identified with a whey aroma, and flavour associated with bitter, sour, and creamy descriptors. It is important to consider the ripening time of three months applied to samples analysed taking into account that Chihuahua cheese is usually consumed within four months of production. As opposed to Cheddar cheese that is consumed after 8-12 months of maturation, Chihuahua cheese is preferred as a semi-matured cheese. The main characteristics appreciated by consumers are its stretchability and melting properties [4]. The rheological profile of Chihuahua cheese has been more related to fresh Colby than to Cheddar or Chester [25].

Previous reports have described Chihuahua cheese with salty, sour, and bitter flavour and diacetyl, cooked, whey as main descriptors for aroma in cheese manufactured with pasteurized milk and with intense notes of sour and bitter in cheese manufactured with raw milk; the authors reported differences among manufacturers [8, 9]. In another report, Chihuahua cheese manufactured with raw milk was described as having characteristics of young, basic cheese with slight bitter notes [5]. On the other hand, Hernández-Morales et al. [2] included the descriptors bitter and sour (mean scores of 2.2 and 3.4, respectively) for Añejo (aged) Mexican cheese; these values are similar to the scores presented in samples of Chihuahua cheese manufactured during summer.

In a recent report by Lopez-Díaz and Martinez-Ruiz [26], the authors describe a sensorial profile from commercial samples of Chihuahua cheese by trained judges and consumer preference tests. They used cheese manufactured in the state of Chihuahua that was obtained from retail supermarkets, but there was no information on their manufacture conditions. Based on the results from consumer preferences, cheese was described by their trained panelists as intense aroma, cooked, butter, and fresh milk aroma descriptors. On the other hand, consumers did not appreciate high acidity, salty taste, weak odour, high-fat content, or bitter taste in Chihuahua cheese. In contrast to the report by Lopez-Díaz and Martínez-Ruiz [26], we tested Chihuahua cheese produced with raw milk, and we have previously reported their microbiological and physicochemical profile [7, 27].

Principal Component Analysis can help on the identification of association not distinguished at first sight of the analysed variables. We used PCA to determine if sampling season or if the fact that Mennonite or non-Mennonite owners operated the dairies can affect the sensorial profile of Chihuahua cheese. The result of plotting the response for the first three components (Figure 2) demonstrated that PC1, PC2, and PC3 explained 90.9% of variance, and three groups were identified (K-means analysis) with higher influence of sampling season on the group formation, rather than if the cheesemaker was either Mennonite or non-Mennonite, or the dairy geographical location. Contrary to what we report here, Olson et al. [28] described that season was not important in the functional properties of Chihuahua cheese; instead, aging or the use of pasteurized or raw milk had an effect on cheese functional properties, although the effect of seasonality was previously reported for rheological characteristics [29].

Mexican sanitary authorities are requesting cheese producers to use pasteurized milk as starter material; therefore, it was important to describe the sensory profile of the few farmers that still manufactured Chihuahua cheese using raw milk and with traditional techniques [1]. The composition of the microbiota responsible for cheese production and ripening has an essential role in the development of a cheese's sensory profile cheese. Although commercial starter cultures are mixed to provide similar cheese characteristics than those presented in traditional manufacturing, cheese manufactured with indigenous bacteria presented stronger flavour descriptors in buffalo and cow milk as compared to commercial cultures [30].

The complex mixture of geographical, seasonal, and environmental characteristics associated with cheese production can influence the particular sensory profile of cheese. In a report on semi-matured and matured Cheddar cheese, consumers were able to differentiate cheeses elaborated in different farms, even when they were only 80 km apart, regardless of the use of pasteurized or raw milk for Cheddar cheese manufacturing [31]. Almanza et al. [6] carried out a consumer preference analysis and demonstrated that the average consumer from Chihuahua City preferred the cheese manufactured in the state of Chihuahua as opposed to cheese elaborated in other Mexican states or even in other countries. All the information related to Chihuahua cheese characteristics, rheological, microbiological, and sensory properties can help on the search for a certificate of origin of denomination, which will help on the preservation of the traditional manufacturing process [3].

## 5. Conclusions

The sensorial analysis of Chihuahua cheese produced in traditional cheese factories, using raw milk as starting material, can be used to create a sensorial profile of the product, which can be used to describe the distinctive characteristics of Chihuahua cheese better. Based on the Quantitative Descriptive Analysis, the sensorial profile of Chihuahua cheese can be described as a semi-matured cheese with a bitter flavor, slightly salted and with a cream flavor, with aroma notes associated with whey and sour; all this related to its short ripening time.

The season of production of Chihuahua cheese had a considerable influence on flavour and aroma characteristics, more than dairies' geographical location.

## Figures and Tables

**Table 1:** Sensory evaluation terms used for evaluation of Chihuahua cheese [13, 14].

**Term**	**Definition**	**Reference**
Cooked	Aromatics associated with cooked milk	Skim milk heated to 85°C for 30 min
Whey	Aromatics associated with fresh whey	Fresh whey
Diacetyl (butyric fat)	Aromatics associated with diacetyl	Diacetyl, 20 ppm
Fruity	Aromatics associated with different fruits	Fresh pineapple, Ethyl hexanoate, 20 ppm
Sulphur	Aromatics associated with sulphurous compounds	Boiled mashed egg
Free fatty acid	Aromatics associated with short-chain fatty acids	Butyric acid 20 ppm
Nutty	The nut-like aromatic associated with different nuts	lightly toasted unsalted nuts, wheat germ, unsalted wheat thins, roasted peanut oil extract
Bitter	Fundamental taste sensation elicited by caffeine, quinine	Caffeine 0.08% in water
Salty	Fundamental taste sensation elicited by salts	Sodium chloride 0.5% in water
Sweet	Fundamental taste sensation elicited by sugars	Sucrose 5% in water
Sour	Fundamental taste sensation elicited by acids	Citric acid 0.08% in water
Umami	Chemical feeling factor elicited by certain peptides and nucleotides	Monosodium glutamate 1% in water
Creamy	Characteristic flavour of cream milk	Cream milk

Concentrated chemical references were prepared in 95% ethanol.

**Table 2:** Effect of dairy and sampling season on the sensory scores of Chihuahua cheese flavour.

	**Terms**												
	Cooked	Whey	Diacetyl	Fruity	Free fatty acid	Sulphur	Nutty	Bitter	Salty	Sweet	Sour	Umami	Creamy
**Dairy**	**Summer**												

**A**	1.5^b^	5.2 ^a^	2.8 ^a^	1.4 ^ab^	3.0 ^a^	1.2 ^a^	1.1 ^a^	6.1 ^a^	3.5 ^a^	1.5 ^a^	3.8 ^a^	3.1 ^a^	3.4 ^abc^

**B**	3.3^a^	3.9 ^ab^	1.4 ^a^	0.9 ^ab^	0.8 ^b^	0.8 ^a^	0.5 ^a^	4.2 ^abc^	1.4 ^bc^	0.6 ^a^	3.8 ^a^	1.6 ^a^	2.8 ^bcde^

**C**	3.1^ab^	3.7 ^ab^	2.3 ^a^	0.6 ^b^	1.8 ^ab^	1.1 ^a^	0.4 ^a^	4.3 ^ab^	1.5 ^bc^	0.6 ^a^	2.9 ^a^	2.2 ^a^	3.5 ^ab^

**D**	2.7 ^ab^	3.3 ^b^	2.4 ^a^	0.6 ^b^	1.5 ^ab^	0.9 ^a^	1.3 ^a^	2.5 ^bc^	2.9 ^abc^	0.8 ^a^	2.0 ^a^	2.1 ^a^	2.0 ^bcde^

**E**	1.9 ^ab^	2.4 ^b^	2.1 ^a^	0.4 ^b^	1.1 ^ab^	1.1 ^a^	0.6 ^a^	2.1 ^c^	2.8 ^abc^	0.9 ^a^	2.4 ^a^	2.6 ^a^	1.1 ^e^

	**Autumn**												

**A**	2.1 ^ab^	3.3 ^b^	1.4 ^a^	1.1 ^ab^	0.8 ^ab^	0.2 ^a^	0.8 ^a^	2.9 ^bc^	1.9 ^abc^	0.3 ^a^	2.0 ^a^	2.3 ^a^	1.4 ^de^

**B**	2.5 ^ab^	2.8 ^b^	1.6 ^a^	1.3 ^ab^	1.1 ^ab^	0.7 ^a^	0.4 ^a^	3.8 ^bc^	1.2 ^c^	0.4 ^a^	2.1 ^a^	1.6 ^a^	2.5 ^bcde^

**C**	2.4 ^ab^	2.6 ^b^	2.3 ^a^	1.5 ^ab^	1.0 ^b^	0.4 ^a^	0.3 ^a^	2.4 ^bc^	1.9 ^abc^	1.0 ^a^	1.9 ^a^	2.1 ^a^	5.1 ^a^

**D**	2.1 ^ab^	2.9 ^b^	1.6 ^a^	2.0 ^a^	0.9 ^ab^	0.3 ^a^	0.3 ^a^	2.5 ^bc^	3.4 ^ab^	1.2 ^a^	2.3 ^a^	2.1 ^a^	3.1 ^bcd^

**E**	1.5 ^b^	3.0 ^b^	1.5 ^a^	1.1 ^ab^	1.0 ^ab^	1.2 ^a^	0.5 ^a^	2.2 ^bc^	2.1 ^abc^	0.8 ^a^	2.0 ^a^	2.5 ^a^	1.5 ^cde^

	**F ratio from ANOVA**												

**Dairy**	4.25*∗∗*	4.81*∗∗*	1.04	1.05	1.60	0.77	1.86	875*∗∗*	6.08*∗∗*	0.97	1.36	1.79	13.02*∗∗*
**Season**	2.68	11.69*∗∗*	4.50*∗*	12.64*∗∗*	5.84*∗*	5.01*∗*	4.98*∗*	13.08*∗∗*	1.60	0.65	9.31*∗∗*	0.47	0.46
**Dairy** **∗** **Season**	1.25	3.37*∗*	1.28	2.72*∗*	2.60*∗*	0.83	1.27	4.57*∗∗*	2.09	2.71*∗*	1.60	0.33	5.61*∗∗*

Values with the same letters in a column indicate that samples do not differ significantly at a significance level of 5%. F values identified with an asterisk show statistical differences at P<0.05, while those identified with two asterisks show differences at P<0.01.

## Data Availability

The data used to support the findings of this study are available from the corresponding author upon request.
